# Fluorescence In Situ Hybridization (FISH) Analysis of the Locations of the Oligonucleotides 5S rDNA, (AGGGTTT)_3_, and (TTG)_6_ in Three Genera of Oleaceae and Their Phylogenetic Framework

**DOI:** 10.3390/genes10050375

**Published:** 2019-05-17

**Authors:** Xiaomei Luo, Juncheng Liu

**Affiliations:** College of Forestry, Sichuan Agricultural University, Wenjiang District, Chengdu 611130, China; juhnson@foxmail.com

**Keywords:** tandem repeat, multicolour FISH, cytomolecular map, telomere, intercalary band

## Abstract

We report the cytogenetic map for a collection of species in the Oleaceae, and test similarities among the karyotypes relative to their known species phylogeny. The oligonucleotides 5S ribosomal DNA (rDNA), (AGGGTTT)_3_, and (TTG)_6_ were used as fluorescence in situ hybridization (FISH) probes to locate the corresponding chromosomes in three Oleaceae genera: *Fraxinus pennsylvanica*, *Syringa oblata*, *Ligustrum lucidum*, and *Ligustrum* × *vicaryi*. Forty-six small chromosomes were identified in four species. (AGGGTTT)_3_ signals were observed on almost all chromosome ends of four species, but (AGGGTTT)_3_ played no role in distinguishing the chromosomes but displayed intact chromosomes and could thus be used as a guide for finding chromosome counts. (TTG)_6_ and 5S rDNA signals discerned several chromosomes located at subterminal or central regions. Based on the similarity of the signal pattern (mainly in number and location and less in intensity) of the four species, the variations in the 5S rDNA and (TTG)_6_ distribution can be ordered as *L. lucidum* < *L.* × *vicaryi* < *F. pennsylvanica* < *S. oblata*. Variations have observed in the three genera. The molecular cytogenetic data presented here might serve as a starting point for further larger-scale elucidation of the structure of the Oleaceae genome, and comparison with the known phylogeny of Oleaceae family.

## 1. Introduction

The Oleaceae family comprises approximately 30 genera and over 600 species in tropical, subtropical and temperate regions of the world, mainly in Asia [1]. Ten genera and more than 160 species (95 endemic) whose chromosome complement is arranged from 2n = 22 to 2n = 52 (e.g., the genera *Myxopyrum* 2n = 22, *Jasminum* 2n = 24/26/39/48/52, *Fontanesia* 2n = 26, *Forsythia* 2n = 28/42, *Chionanthus* 2n = 46, *Osmanthus* 2n = 46, *Olea* 2n = 46, *Fraxinus* 2n = 46, *Ligustrum* 2n = 46, and *Syringa* 2n = 46/48) are distributed in China [2,3,4]. The most common chromosome base number in Oleaceae is x = 23 [5]. To date, determination of the location of a chromosome through fluorescence in situ hybridization (FISH) has only been reported in one species, *Olea europaea* ssp. *sativa* in Oleaceae; in this species, Katsiotis et al. [6] identified two tandemly repeated DNA sequences in the chromosomes of this species: the 81-bp family and pOS218 co-localization. In addition, Jeandroz et al. [7] constructed physical maps of *Fraxinus excelsior* and *F. oxyphylla* by Southern blotting (18S, 25S, and 35S ribosomal DNA (rDNA)). Although only approximately five genetic maps of Oleaceae, including several integrated maps with a higher marker density [8,9,10,11,12], have been published, these constitute a foundational tool and a resource for marker-assisted selection and genomic studies. However, the linkage maps provide little information about the physical locations, distribution, distances, and orientation of genetic markers. Cytogenetic maps encompassing the information from both genetic and cytological maps can relate the markers mapped across linkage groups to the cytological position on chromosomes. Until recently, the cytogenetic maps of Oleaceae trees have rarely been studied due to their small chromosomes, which are less than 5 μm [13], and the lack of genome information for Oleaceae species. Information about evolution and speciation can be obtained by studying the molecular and genomic organization of repetitive sequences. Furthermore, the physical localization of such repetitive sequences can provide information about the structure of the Oleaceae chromosomes, which have not been previously described cytologically.

Mass repetitive DNA sequences comprise half of tree genomes [14], which can be organized in either tandem repeat DNA sequences or a dispersed manner. The former can vary in length from two to many thousands of base pairs [15,16]. Numerous studies on smaller repeat DNA sequences in plants have been performed over the last few decades, and detailed information about the evolution and differences among related species has been obtained from physical maps [17,18,19]. The DNA content of six Oleaceae genera, e.g., *Fraxinus* 0.87–0.99 pg, *Syringa* 1.20–1.30 pg, *Nyctanthes* ~1.23 pg, *Jasminum* ~1.44 pg, *Olea* 1.50–2.99 pg, and *Ligustrum* ~1.57 pg [20,21,22,23,24,25,26], has been estimated in the Plant DNA C-values Database [27]. The repetitive DNA of these Oleaceae genera has been defined, and the genome size (1 pg ≈ 978 Mb) varies among genera.

Repetitive DNA is the predominant component of heterochromatin and is typically associated with the centromeric, pericentromeric, subtelomeric and telomeric regions of chromosomes [28,29,30,31,32,33,34,35,36]. The telomere repeats (CCCTAAA)_n_, (TTTAGGG)_n_, (TTAGG)_n_, and (AGGGTTT)_n_, which are found in most angiosperms, have been localized to the ends of the gymnosperm chromosome. *Picea abies* (L.) Karsten and *Larix decidua* Mill. exhibit only terminal sites, but hybridization at centromeric sites has also been observed in *Pinus sylvestris* L. [37,38] and several intercalary sites in *Pinus elliottii* Engelm. [39]. The presence of interstitial telomeric signals on chromosomes has been correlated with chromosome rearrangements; as a result, these have been used as markers of chromosome evolution and could therefore be used to compare the phylogenetic relationship of species and even populations [40,41]. Trinucleotide (TTG)_6_ is most often repeated in the 4D-genome chromosomes of *Avena* species [42] and is also located in the pericentromeric and, occasionally, telomeric chromosome regions but exhibits low matching to the C genome of the *Avena* species [43]. This (TTG)_6_ probe has also shown signals at the pericentromeric chromosome regions of *Hippophae rhamnoides* L. (unpublished work). The ribosomal DNA (rDNA), such as 5S rDNA, have been physically mapped to the chromosome of plant species [44,45] and are useful for understanding the general patterns of chromosome evolution among related species and for cytotaxonomic approaches [46,47,48]. The physical chromosome maps for the Oleaceae genera *Fraxinus*, *Syringa*, and *Ligustrum* remain unknown because no repetitive sequence-based FISH studies have yet been performed.

Because the chromosome count (2n = 46) and genome size vary among the genera *Fraxinus*, *Syringa*, and *Ligustrum*, the differences in their genome structure remain unknown. Thus, this study addresses the lack of knowledge on the physical locations of the repetitive elements oligo-5S rDNA, (AGGGTTT)_3_, and (TTG)_6_ on chromosomes from *Fraxinus pennsylvanica*, *Syringa oblata*, *Ligustrum lucidum*, and *Ligustrum* × *vicaryi* through a FISH analysis. As a result, the present work increases the available knowledge on the structure of the Oleaceae genome, provides cytogenetic data for the identification of a number of individual chromosomes, and comparison with the known phylogeny of Oleaceae family.

## 2. Materials and Methods

### 2.1. Seed Materials and Root Tips

Oleaceae plants are widely used as ornamentals on university campuses in China. Seeds of *Fraxinus pennsylvanica* Marsh., *Syringa oblata* Ait., *Ligustrum lucidum* Lindl., and *Ligustrum* × *vicaryi* Rehder (= *Ligustrum ovalifolium* Hassk. var. *aureomarginatum* Hort. Ex Rehd × *Ligustrum vulgare* Linn.) were collected from the Chengdu Campus of Sichuan Agricultural University, germinated in wet sand pots and placed at room temperature under natural light conditions. Once the roots reached 1.5–2.0 cm, the root tips were excised and soaked in nitrous oxide for 4 h. After this treatment, the root tips were placed in glacial acetic acid for 5 min and then maintained in 75% ethyl alcohol at −20 °C until use.

### 2.2. Chromosome Preparation

The ethyl alcohol on the root tips was washed off using ddH_2_O, and the meristems were then cut off, immediately transferred into a mixture of cellulase and pectinase (2:1) and maintained in this mixture at 37 °C for 45 min. After this treatment, the enzyme mixture on the meristems was washed off using ddH_2_O, the ddH_2_O was washed off with ethyl alcohol, and all the ethyl alcohol was subsequently removed. After the meristems were air dried, 20 μL of glacial acetic acid was added to each meristem to prepare a suspension, and 10 μL of the mixture was dropped onto one clean slide. The slides were air dried and examined using an Olympus CX23 microscope (Olympus Corporation, Tokyo, Japan). The well-spread metaphase chromosomes were used for further in situ hybridization experiments.

### 2.3. Probe Preparation

The chromosome end repeat sequence (AGGGTTT)_3_ [49], the ribosome DNA sequence 5S rDNA [36], and the trinucleotide sequence (TTG)_6_ [43] were used in this study. These oligonucleotide sequences were produced by Sangon Biotechnology Co., Ltd. (Shanghai, China), and the 5’ ends were labelled with 6-carboxyfluorescein (FAM) or 6-carboxytetramethylrhodamine (TAMRA). The synthetic probes were dissolved in 1 × Tris - Ethylene Diamine Tetraacetic Acid (TE) and maintained at a concentration of 10 μM at −20 °C until use.

### 2.4. FISH Hybridization

The slides with well-spread metaphase chromosomes were fixed in 4% paraformaldehyde for 10 min, shaken twice with 2 × saline sodium citrate (SSC) buffer for 5 min and subjected to 5-min incubations with 75%, 95%, and 100% ethyl alcohol. After the slides were air dried, 60 μL of 70% deionized formamide (FA) was dropped onto the chromosomes, and coverslips (24 cm × 24 cm) were placed in 70% FA at 80 °C for 2 min. After this treatment, the coverslips were immediately removed from the slides and discarded, and the slides were then subjected to 5 min incubations in 75%, 95%, and 100% ethyl alcohol (precooled at −20 °C). Ten microliters of hybridization solution, which included 0.35 μL of each probe, 4.825 μL of 2 × SSC, and 4.475 μL of 1 × TE, was dropped onto the chromosomes, and coverslips (24 cm) were placed on top of the hybridization solution. The slides were then incubated for 2 h at 37 °C.

### 2.5. Image Capture and Analysis

After hybridization, the slides were shaken and washed with 2 × SSC buffer to remove the coverslips. Ten microliters of 4,6-diamidino-2-phenylindole (DAPI) was dropped onto the air-dried chromosomes, and coverslips (24 cm × 24 cm) were placed on top of the DAPI solution. The slides were examined using an Olympus BX63 fluorescence microscope combined with a Photometric SenSys Olympus DP70 CCD camera (Olympus Corporation, Tokyo, Japan).

Approximately 10 well-separated metaphase chromosomes were observed from each species to determine the chromosome number. The three best spreads were used for analysis of the signal patterns. The length of each chromosome was calculated using Photoshop version 7.1 (Adobe Systems Inc., San Jose, CA, USA), and each spread was measured three times to obtain consistent chromosome data. The chromosomes were arranged by length from longest to shortest.

## 3. Results

### 3.1. Chromosome Number and Size

**Figure 1:**
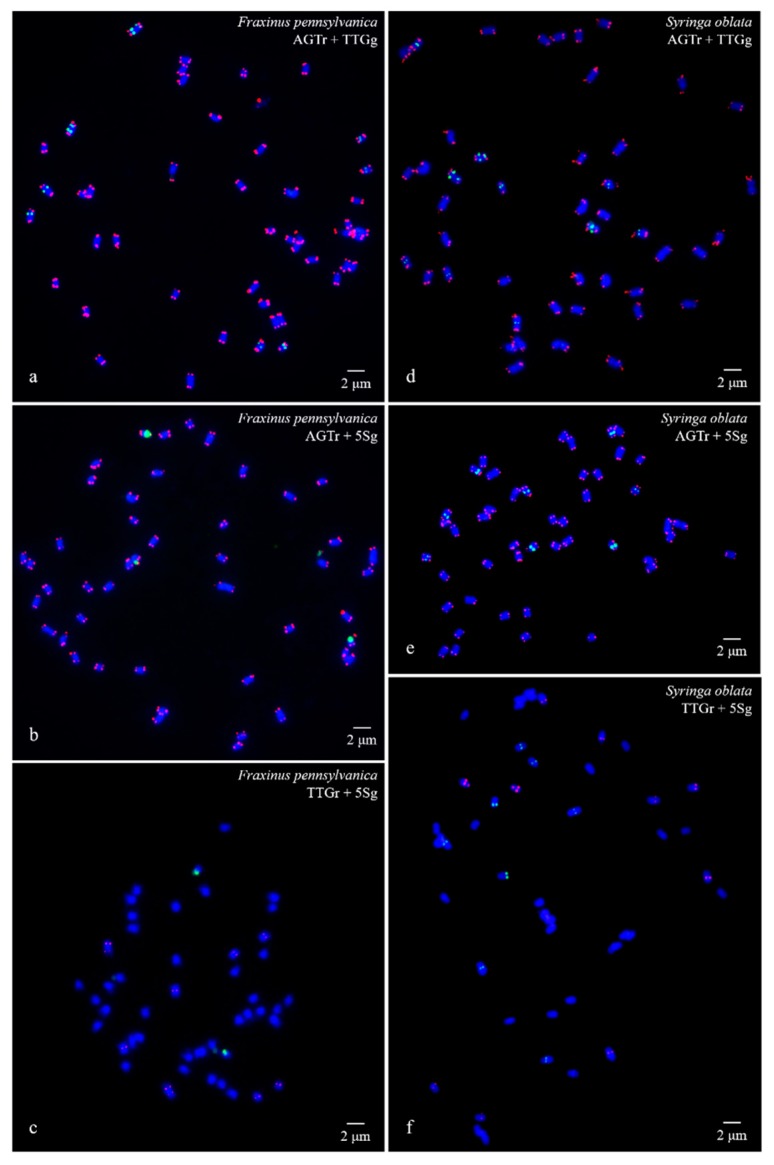
Visualization of mitotic metaphase chromosomes of *Fraxinus pennsylvanica* (**a**–**c**) and *Syringa oblata* (**d**–**f**) after fluorescence in situ hybridization (FISH). The first probe of (AGGGTTT)_3_ was labelled with 6-carboxytetramethylrhodamine (TAMRA) (red) in (**a**), (**b**), (**d**), and (**e**) (abbreviation AGTr); the second probe of 5S rDNA was labelled with 6-carboxyfluorescein (FAM) (green) in (**b**), (**c**), (**e**), and (**f**) (abbreviation 5Sg); and the third probe of (TTG)_6_ was labelled with 6-FAM (green) in a and d (abbreviation TTGg) and with TAMRA (red) in (**e**) and (**f**) (abbreviation TTGr). The concentration of all the probes was 10 μM. All the chromosomes were counterstained with 4,6-diamidino-2-phenylindole (DAPI) (blue). Scale bar = 2 μm.

**Figure 2:**
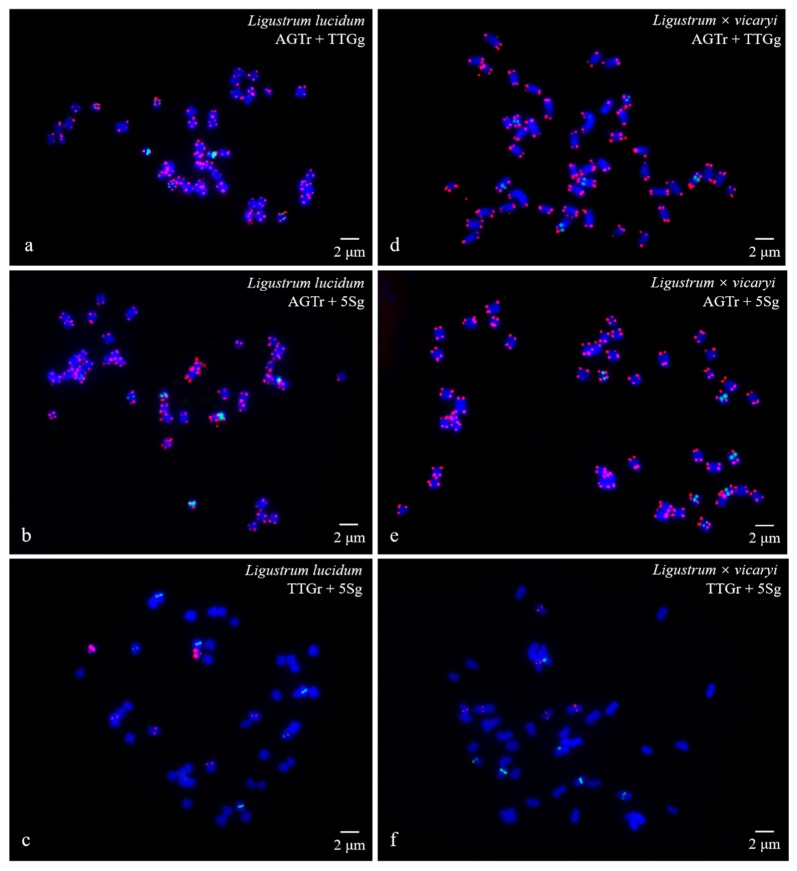
Visualization of mitotic metaphase chromosomes of *Ligustrum lucidum* (**a**–**c**) and *Ligustrum × vicaryi* (**d**–**f**) after fluorescence in situ hybridization (FISH). The first probe of (AGGGTTT)_3_ was labelled with TAMRA (red) in (**a**), (**b**), (**d**), and (**e**) (abbreviation AGTr); the second probe of 5S rDNA was labelled with 6-FAM (green) in (**b**), (**c**), (**e**), and (**f**) (abbreviation 5Sg); and the third probe of (TTG)_6_ was labelled with 6-FAM (green) in a and d (abbreviation TTGg) and with TAMRA (red) in (**e**) and (**f**) (abbreviation TTGr). The concentration of all the probes was 10 μM. All the chromosomes were counterstained with 4,6-diamidino-2-phenylindole (DAPI) (blue). Scale bar = 2 μm.

**Figure 3:**
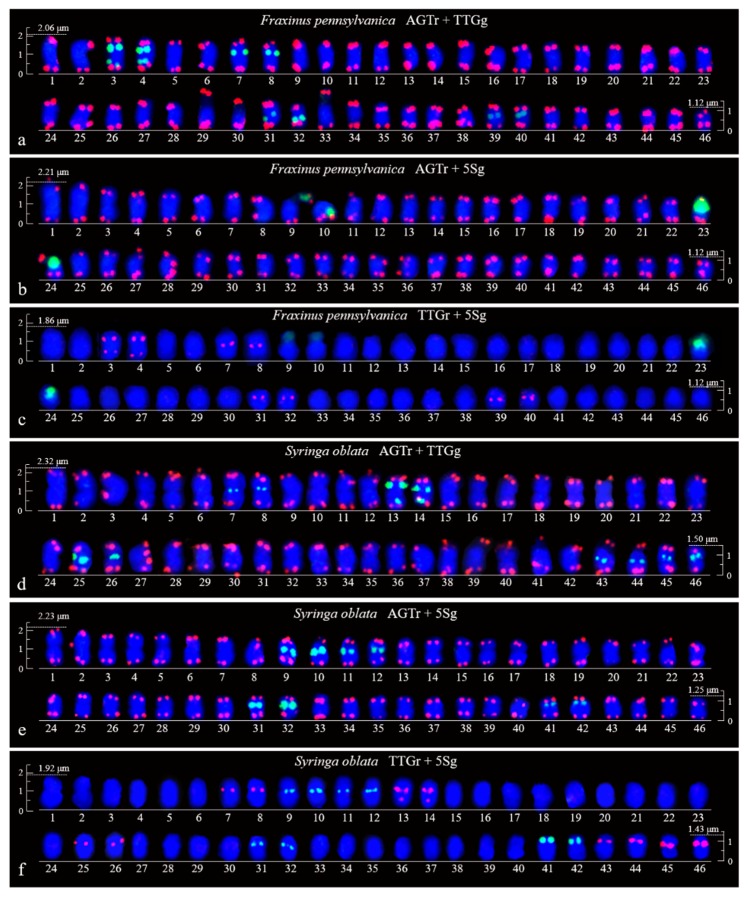
Aligned chromosomes of *Fraxinus pennsylvanica* (**a**–**c**) and *Syringa oblata* (**d**–**f**) captured from Figure 1a–f. The chromosome alignments are based on the length, i.e., from the longest (No. 1) to the shortest chromosome (No. 46). The chromosome numbers are only approximate due to their small size. Scale bars are placed at the beginning and end of each chromosome alignment (2.5–1.5 μm).

**Figure 4:**
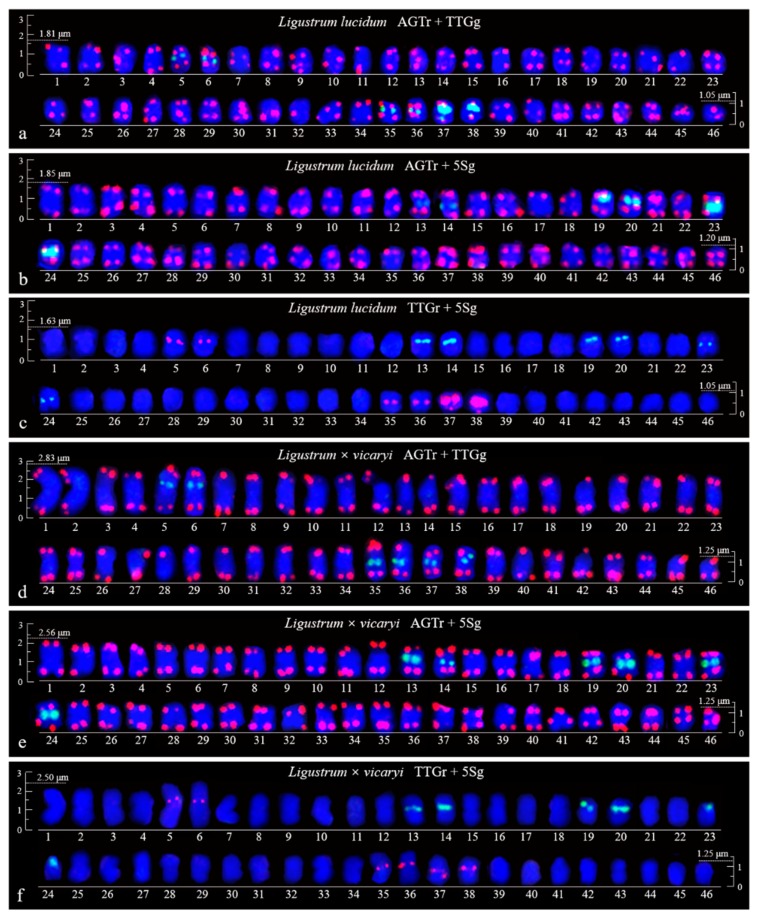
Aligned chromosomes of *Ligustrum lucidum* (**a**–**c**) and *Ligustrum × vicaryi* (**d**–**f**) captured from Figure 2a–f. All the chromosome alignments (with the exception of that shown in Figure 4d,f) are based on the length, i.e., from the longest (No. 1) to the shortest chromosome (No. 46). The chromosome numbers are only approximate due to their small size. Scale bars are placed at the beginning and end of each chromosome alignment (3.0–1.5 μm).

FISH images of mitotic metaphases of *Fraxinus pennsylvanica*, *Syringa oblata*, *Ligustrum lucidum*, and *Ligustrum* × *vicaryi* are illustrated in Figure 1 and Figure 2. Chromosomes in three spreads of each species were labelled with either of the two probes for (AGGGTTT)_3_, (TTG)_6_ and 5S rDNA. Forty-six chromosomes were counted in all four species. Karyogram reconstructions from the in situ hybridization images are illustrated in Figure 3 and Figure 4. The chromosome lengths from three metaphases of *F. pennsylvanica* (range from 2.06–1.12 μm, 2.21–1.12 μm, 1.86–1.12 μm), *S. oblata* (range from 2.32–1.50 μm, 2.23–1.25 μm, and 1.92–1.43 μm), *L. lucidum* (range from 1.81–1.05 μm, 1.85–1.20 μm, and 1.63–1.05 μm), *L. × vicaryi* (range from 2.83–1.25 μm, 2.56–1.25 μm, and 2.50–1.25 μm) were calculated. A very significant difference in the chromosome size was found among the four species (p value = 0.00187). All 12 metaphase chromosome lengths ranged from 2.83 μm to 1.05 μm, which indicated that these were small. The species were ordered in terms of chromosome length as *L. lucidum* < *F. pennsylvanica* < *S. oblata* < *L.* × *vicaryi*. Undistinguishable centromeres make it difficult to determine the long and short arms, and chromosomes with a small and similar size make it difficult to match the chromosome pair.

### 3.2. Probe Signal Distribution

(AGGGTTT)_3_ signals were observed in almost all chromosome ends of the four species (Figure 1a,b,d,e; Figure 2a,b,d,e; Figure 3a,b,d,e; Figure 4a,b,d,e). (TTG)_6_ signals (eight strong and two weak signals) were observed in the subterminal region of both arms of two chromosomes and in the central region of six chromosomes in *F. pennsylvanica* (Figure 1a,c; Figure 3a,c). (TTG)_6_ signals (six strong and six relatively weak signals) were observed in the subterminal region of both arms of two chromosomes and in the central region of eight chromosomes in *S. oblata* (Figure 1d,f; Figure 3d,f). (TTG)_6_ signals (two strong and six relatively weak signals) were observed in the subterminal region of both arms of two chromosomes and in the central region of four chromosomes in *L. lucidum* (Figure 2a,c; Figure 4a,c). (TTG)_6_ signals (six relatively strong signals) were observed in the central region of six chromosomes in *L.* × *vicaryi* (Figure 2d,f; Figure 4d,f). 5S rDNA signals (two large strong and two weak signals) were observed in the subterminal region of four chromosomes in *F. pennsylvanica* (Figure 1b,c; Figure 3b,c). In addition, 5S rDNA signals (six relatively strong signals) were observed in the subterminal region of two chromosomes and in the central region of six chromosomes in *S. oblata* (Figure 1e,f; Figure 3e,f), and 5S rDNA signals (six relatively strong signals) were observed in the subterminal region of two chromosomes and in the central region of four chromosomes in *L. vulgare* (Figure 2b,c; Figure 4b,c) and in *L.* × *vicaryi* (Figure 2e,f; Figure 4e,f).

### 3.3. Phylogenetic Comparisons

**Figure 5:**
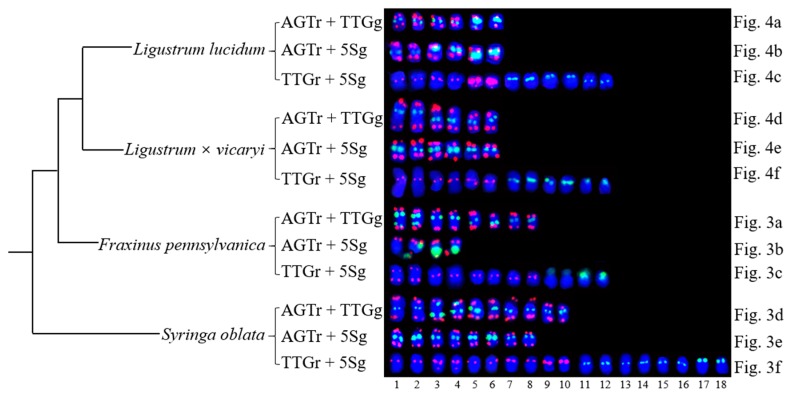
The variations of the (AGGGTTT)_3_, 5S rDNA and (TTG)_6_ distribution in Oleaceae (three genera) sorted from the data shown in Figure 3 and Figure 4. Each probe combination of each species is annotated on the left of each chromosomal pattern. Figure 5 only exhibits chromosomes with AGTr + TTGg signals, or AGTr + 5Sg with signals, or TTGr + 5Sg signals, while Figure 5 do not present chromosomes with no signals, or only with AGTr signals. Therefore, Figure 5 is a simplified version of Figure 3 and Figure 4. To explicit correspond each chromosomal pattern between Figure 3, Figure 4. and Figure 5, Figure 3a–f, Figure 4a–f were annotated on the right of each chromosomal pattern in Figure 5. The bottom numbers represent chromosome line number. The chromosome alignment is similar to the chromosome in Figure 3 and Figure 4.

Because the (AGGGTTT)_3_ signals showed little difference among the chromosomes and the (TTG)_6_ signals and 5S rDNA signals discerned several chromosomes, as demonstrated in Figure 3 and Figure 4, chromosomes bearing (TTG)_6_ and 5S rDNA signals were arrayed together in Figure 5. The signal pattern obtained from the probe combinations (AGTr + TTGg, AGTr + 5Sg, and TTGr + 5Sg) showed similarity (major similarity in number and location and minor similarity in intensity) between *L. lucidum* and *L.* × *vicaryi*, and the two *Ligustrum* species shared central (TTG)_6_ signals from four chromosomes, subterminal 5S signals from two chromosomes and central 5S rDNA signals from four chromosomes, indicating a close relationship. (TTG)_6_ signals from *F. pennsylvanica* chromosomes were obtained from two additional signal sites, but two fewer 5S rDNA signals were obtained from the chromosomes of this species; thus, the total number of signals from *F. pennsylvanica* was equal to that from the two *Ligustrum* species, indicating a relatively close relationship. Compared with the signal numbers from *Ligustrum* species, four more (TTG)_6_ signals were obtained from the *S. oblata* chromosomes, and two more 5S rDNA signals were obtained from these chromosomes, indicating a relatively distant relationship. The variations in the 5S rDNA and (TTG)_6_ distribution can be ordered as *L. lucidum* < *L.* × *vicaryi* < *F. pennsylvanica* < *S. oblata*. Variations have observed in the three Oleaceae genera.

## 4. Discussion

The present study is the first to use oligo-5S rDNA, (AGGGTTT)_3_, and (TTG)_6_ to locate chromosomes from three genera in Oleaceae and thereby compare with the known phylogeny. This work improves the understanding of the organization of Oleaceae chromosomes at a cytogenetic level. The following discussion refers to 1) the variation in the chromosome size, 2) the role of oligo-5S rDNA, (AGGGTTT)_3_, and (TTG)_6_, and 3) comparison with the known phylogeny of related genera from the Oleaceae family.

### 4.1. Variation in Chromosome Size

It is impossible to identify many karyotypic features of mitotic metaphase chromosomes of species with small chromosomes, such as *F. pennsylvanica*, *S. oblata*, *L. lucidum*, and *L.* × *vicaryi*. Therefore, in these cases, a karyotype analysis only shows the chromosome number and length. The chromosome numbers obtained in this study were all in accordance with previously published data [2,3,4]. In the present study, the species were ordered based on the chromosome length as *L. lucidum* < *F. pennsylvanica* < *S. oblata* < *L.* × *vicaryi*. Nevertheless, the species could be ordered as *Fraxinus* < *Syringa* < *Ligustrum* based on the genome size detailed in the Plant DNA C-values Database [27]. The explanations underlying the different chromosome sizes of *Ligustrum* species are likely the incomplete simultaneous phases of cell division, unrepresentative C-values for the measured species in *Ligustrum*, or differential accumulation of transposable elements [50,51,52].

### 4.2. Roles of Oligo—(AGGGTTT)_3_, 5S rDNA, and (TTG)_6_

A FISH analysis of species belonging to three genera has not been previously performed. The FISH analysis of four species using the (AGGGTTT)_3_ probe revealed signals from almost all chromosome ends, which showed that the chromosomes were intact and guided the counting of the chromosome number based on number of (AGGGTTT)_3_ signal sites. In previous studies, (AGGGTTT)_3_ signals were usually observed at the terminal position, but some signals have also occasionally been detected at the pericentromeric and internal positions [19,53,54,55,56,57]. Several studies have shown that the presence of non-telomeric signals or interstitial telomeric signals can indicate that the chromosomes have undergone structural and/or numerical rearrangements. No interstitial telomere sequences were detected on the chromosomes of *F. pennsylvanica*, *S. oblata*, *L. lucidum*, and *L.* × *vicaryi*. Thus, it could be hypothesized that no chromosome rearrangements caused by a type of chromosome fusion with telomeric sequence footprints have occurred in these four species, and the chromosomes observed in these species might represent the ancestral chromosomes of Oleaceae. Nevertheless, we did not ignore the fact that small interstitial telomeric signals originating from chromosomal fusions were not detected due to the limited resolution of the FISH technique.

The most unique 5S rDNA pattern was found in *F. pennsylvanica*. This species contained the fewest 5S rDNA sites, but the signal locations of these sites were noticeably more adjacent to the base (AGGGTTT)_3_ signals than those of other species, which indicated movement of the 5S rDNA repeats within each site. The reported cytogenetic data support a close-to-basal position for *F. pennsylvanica* [19,58,59]. 

Diversity in 5S rDNA and (TTG)_6_ sites among closely related species often characterizes diploids and their polyploid relatives [43,60,61]. A previous study on *Paphiopedilum*, an orchid genus with no known polyploids [62], also found high variation in the number and distribution of the 5S rDNA sites among close relatives, and the researchers explained these findings by chromosomal rearrangements and dynamic double-strand break repair processes that characterize hotspots in pericentromeric and telomeric regions [63]. These findings could also be observed in this study of four species with no known polyploids (x = 23) and with diverse sites. A plausible explanation for the observed variations in the chromosome distribution is the rapid amplification and/or reduction of repetitive elements [31,32].

### 4.3. Phylogenetic Comparisons

Variations in the 5S rDNA and (TTG)_6_ distribution have a phylogenetic framework because the closeness of taxa is correlated with the similarity of their signal FISH patterns [19,43,58,64,65]. Among the four species of Oleaceae examined here, the 5S rDNA and (TTG)_6_ site patterns of the two *Ligustrum* species *L. lucidum* and *L.* × *vicaryi* showed the highest similarity. In contrast, *F. pennsylvanica* showed decreased and increased divergence in the 5S rDNA and (TTG)_6_ sites, respectively, from *Ligustrum*, and *S. oblata* showed increased divergence from these species in terms of both the 5S rDNA and the (TTG)_6_ sites. This phylogenetic framework (*L. lucidum* < *L.* × *vicaryi* < *F. pennsylvanica* < *S. oblata*) does not agree with those obtained in previous studies. Wang et al. [66] performed an amplified fragment length polymorphism (AFLP) analysis of 17 species of the Oleaceae family and found that the following relationship: *S. oblata* < *L.* × *vicaryi* < *L. lucidum* < *F. pennsylvanica*. Through an analysis of morphological differences, Chang et al. [3] revealed the following relationship: *L. lucidum* < *L.* × *vicaryi* < *S. oblata* < *F. pennsylvanica*. For example, *S. oblate* and *F. pennsylvanica* have samara or capsule fruits, whereas *L. vicaryi* and *L. lucidum* have berry or berrylike fruits. *L. vicaryi* and *S. oblate* are shrubs, whereas *L. lucidum* and *F. pennsylvanica* are trees. A possible reason for these inconsistent findings is the use of different methods: physical map (FISH), genetic map (AFLP), and morphological characteristics. Furthermore, the different origins and hybridization conditions might affect the relationship of these species. *S. oblate* and *L. lucidum* originate from China, whereas *L. vicaryi* and *F. pennsylvanica* originate from America. In addition, only *L. vicaryi* is a hybrid; specifically, this species is a hybrid of *Ligustrum ovalifolium* Hassk. var. *aureomarginatum* Hort. Ex Rehd × *Ligustrum vulgare* Linn. [67]. Moreover, the available information is quite limited. We need more probes and markers to annotate the maps, and this additional information will more precisely compare with the known phylogeny relationships among Oleaceae genera.

In the present study, the comparison with the known phylogeny was based on two discerning FISH probes, namely, 5S rDNA and (TTG)_6_, but these probes were not combined with chromatin fibre-FISH, molecular genetic mapping, or phylogeny based on plastid, nuclear, and mitochondrial sequences [56,68,69]. Furthermore, the visible fluorescence signal was determined through experiments (e.g., different probe concentrations and hybridization durations) based on capturing the image signals and adjustment of the image signal contrast ratio, among other experimental techniques; hence, it is difficult to obtain coinciding results. Hence, cytogenetics do not provide robust markers for species-wide phylogenetic comparisons, which require combination with effective molecular data.

## 5. Conclusions

At the molecular cytogenetic level, our FISH results highlight both variable and constant features of the signal pattern. The location and number of signal sites in *Fraxinus*, *Syringa*, and *Ligustrum* showed variations. The molecular cytogenetic data presented here might serve as a starting point for further larger-scale elucidation of the structure of the Oleaceae genome, and compare with the known phylogeny of Oleaceae family in the future.
